# External validation of the performance of commercially available deep-learning-based lung nodule detection on low-dose CT images for lung cancer screening in Japan

**DOI:** 10.1007/s11604-024-01704-2

**Published:** 2024-11-30

**Authors:** Wataru Fukumoto, Yuki Yamashita, Ikuo Kawashita, Toru Higaki, Asako Sakahara, Yuko Nakamura, Yoshikazu Awaya, Kazuo Awai

**Affiliations:** 1 Department of Diagnostic Radiology, Graduate School of Biomedical and Health Science, Hiroshima University, 1-2-3 Kasumi, Minamiku, Hiroshima 734-8551 Japan; 2 School of Medicine, Hiroshima University, 1-2-3 Kasumi, Minamiku, Hiroshima 734-8551 Japan; 3 Graduate School of Advanced Science and Engineering, Hiroshima University, 1-4-1 Kagamiyama, Higashi-Hiroshima, 739-8527 Japan; 4 Department of Respiratory Medicine, Miyoshi Central Hospital, 10531 Higashi-Sakaya-cho, Miyoshi, Hiroshima 728-8502 Japan

**Keywords:** Artificial intelligence, Computed-aided detection, Lung cancer screening, Lung nodule, Atypical pulmonary cyst

## Abstract

**Purpose:**

Artificial intelligence (AI) algorithms for lung nodule detection have been developed to assist radiologists. However, external validation of its performance on low-dose CT (LDCT) images is insufficient. We examined the performance of the commercially available deep-learning-based lung nodule detection (DL-LND) using LDCT images at Japanese lung cancer screening (LCS).

**Materials and methods:**

Included were 43 patients with suspected lung cancer on LDCT images and pathologically confirmed lung cancer. The reference standard for nodules whose diameter exceeded 4 mm was set by a radiologist who referred to the reports of two other radiologists reading the LDCT images. After we applied commercially available DL-LND to the LDCT images, the radiologist reviewed all nodules detected by DL-LND. When he failed to identify an existing nodule, it was also included in the reference standard. To validate the performance of DL-LND, the sensitivity for lung nodules and lung cancer, the positive-predictive value (PPV) for lung nodules, and the mean number of false-positive (FP) nodules per CT scan were recorded.

**Results:**

The radiologist detected 97 nodules including 43 lung cancers and missed 3 solid nodules detected by DL-LND. A total of 100 nodules was included in the reference standard. DL-LND detected 396 nodules including 40 lung cancers. The sensitivity for the 100 nodules was 96.0%; the PPV was 24.2% (96/396). The mean number of FP nodules per CT scan was 7.0; sensitivity for lung cancer was 93.0% (40/43). DL-LND missed three lung cancers; 2 of these were atypical pulmonary cysts.

**Conclusion:**

We externally verified that the sensitivity for lung nodules and lung cancer by DL-LND was very high. However, its low PPV and the increased FP nodules remains a serious drawback of DL-LND.

## Introduction

Lung cancer remains one of the leading causes of global cancer-related mortality [1]. More than 75,000 individuals die annually of lung cancer in Japan. The 5-year survival rate is as low as 45.1% according to the annual survival report of hospital-based cancer registries from information service of national cancer center in Japan [2]; for early-stage lung cancer it is 81.9% due to improved treatments. Therefore, early detection and treatment, and primary prevention, e.g. smoking cessation, are essential to reduce the mortality rate from lung cancer.

Randomized controlled lung cancer screening (LCS) trials using low-dose CT (LDCT) studies, including the National Lung Screening Trial (NSLT) and the Dutch-Belgian randomized lung cancer screening (NELSON) trial, reduced the mortality rate by 20–24% compared to chest radiography or no screening in especially high-risk groups [3, 4]. Consequently, as millions of individuals undergo LCS with LDCT every year to reduce lung cancer mortality, the workload of radiologists is overwhelming [5]. Their missing of nodules is one of the most significant problems in LCS. The increased image noise on low-dose and thin-slice images can hamper nodule detection [6]. Indeed, in the large NLST, 6.2% of lung cancer were missed by radiologists [7]. Artificial intelligence (AI), including machine learning and deep-learning, is expected to solve these problems.

AI algorithms for lung nodule detection, i.e. computer-aided detection (CAD), have been developed to assist radiologists. On public databases of Lung Image Database Consortium and Image Database Resource Initiative (LIDC/IDRI), AI algorithms exhibited high sensitivity (83–97%) for lung nodule detection and deep-learning-based algorithms outperformed radiologists especially with respect to small nodules [5, 8]. However, clinically they are not widely used because their false-positive (FP) rate was higher than that of radiologists. Al Mohammad B et al. [9] documented 15.1 FP nodules per scan inspected by radiologists. Besides, the reported performance of AI algorithms for lung nodule detection can vary substantially because different data sets, including the image quality, scan conditions, and vendors, were used for training and evaluation [10]. Thin slices (1 mm or less) are recommended for LDCT used at LCS [11]. As the image noise is increased, the performance of AI algorithms may be affected.

We externally evaluated the performance of commercially available deep-learning-based lung nodule detection (DL-LND) using LDCT images at Japanese LCS.

## Materials and methods

This retrospective study was approved by our institutional review board; prior informed consent was waived.

### Subjects

We enrolled a total of 10,217 subjects who had undergone LCS with LDCT in our institute between 2015 and 2022. Our entry criteria are based on the National Comprehensive Cancer Network (NCCN) guidelines (ver. 1.2013); they included subjects between 55 and 74 years and a history of smoking at least 30 packs of cigarettes a year before smoking cessation less than 15 years prior to enrollment. Alternatively, the entry criteria were an age of at least 50 years, a history of smoking 20 packs per year, and one additional risk factor other than second-hand smoke. Among the 10,217 subjects, 43 were patients with suspected lung cancer on LDCT images and pathologically diagnosed as lung-cancer positive; all but one were males. The median age was 72 years (range 57–78 years). The median smoking index was 940 (range 460–4080). Of the 43 lung cancers, 32 were pathologically diagnosed by surgery and 11 by bronchoscopy. The cancers were 27 adenocarcinomas (6 adenocarcinoma in situ, 3 minimally invasive adenocarcinomas, 17 invasive adenocarcinomas; 1 was only cytologically diagnosed), 13 were squamous cell-, 2 small cell-, and 1 a large-cell carcinoma. Stage 0 was recorded in 6 patients, Stage I in 32, stage II in 1, stage III in 3, and stage IV in 1 patient.

### LDCT scanning

All subjects were scanned with a 320-detector CT scanner (Aquilion ONE, Canon Medical Systems) without contrast material. The scanning parameters for LDCT were helical scans; pitch 1.388, detector configuration 0.5 mm × 80, 120 kV, 25 mAs (50mAs in 2015). The computed tomography dose index (CTDIvol) was 1.5 mGy (3.0 mGy in 2015). The images were reconstructed at a slice thickness/interval of 1.0/1.0 mm (2.0/2.0 mm in 2015), a matrix size of 512 × 512, hybrid iterative reconstruction with lung kernel (AIDR 3D Standard FC51). The image noise, determined as the standard deviation (SD) of the attenuation value in a single circular 100 mm^2^ region of interest placed in the ascending aorta was measured on LDCT images. The mean and SD of the image noise on LDCT images were 39.5 and 3.5 Hounsfield units (HU) (63.9 and 14.0 HU in 2015).

### Reference standards

The reference standard for the nodules whose diameter exceeded 4 mm was determined by a board-certified radiologist with 15 years of experience who referred to the reports of two other board-certified radiologists reading LDCT images. The 4-mm diameter cutoff size was set based on Lung-RADS 2022 and earlier studies [5, 12]. The diameter, subtype, specific lobe, and location of nodules were also recorded. For the location, the peripheral one-third of the lung was defined as peripheral, the hilum of one-third of the lung as the hilum, and the area between them as the intermediate area. For the measurement of nodules less than 10 mm, the average of the long- and short-axis diameters was adopted. For nodule diameters larger than 10 mm, the maximum diameter was adopted [13]. To classify the nodule subtypes based on Lung-RADS 2022, they were recorded as solid nodule, part solid nodule, non-solid nodule (ground glass nodules, GGN) and atypical pulmonary cyst (APC).

### Deep-learning-based lung nodule detection (DL-LND)

We applied the commercially available DL-LND attached to the SYNAPSE SAI viewer V2.4 (FUJIFILM Medical Co. Ltd) on the LDCT images. The recommended subjects and the CT parameters for DL-LND were adults; acquired were non-contrast chest CT images (matrix size 512 × 512, slice thickness 5 mm, slice interval 5 mm, lung kernel). The DL-LND instrument was designed to detect solid nodules larger than 3 mm and sub-solid nodules (part solid nodules and GGNs) larger than 5 mm during the training phase, the sizes could not be changed.

The board-certified radiologist reviewed all detected nodules detected by DL-LND. When he failed to identify a nodule whose diameter exceeded 4 mm it was recorded and included in the reference standard.

### Validation of the performance of DL-LND

To examine the performance of DL-LND, its sensitivity for lung nodules and pathologically confirmed lung cancer, the positive-predictive value (PPV) for lung nodules, and the mean number of FP nodules per CT scan were recorded. The characteristics of FP nodules (perifissural shadows, e.g. perifissural nodules and perivascular nodules, e.g. micro-mucus plugs) were also recorded. The sensitivity for lung nodules was their percentage detected by DL-LND in the reference standard. The PPV for lung nodules was their percentage detected by DL-LND that met the reference standard. The sensitivity for lung cancer was the percentage of lung cancer detected by DL-LND among all lung cancers.

## Results

**Fig. 1:**
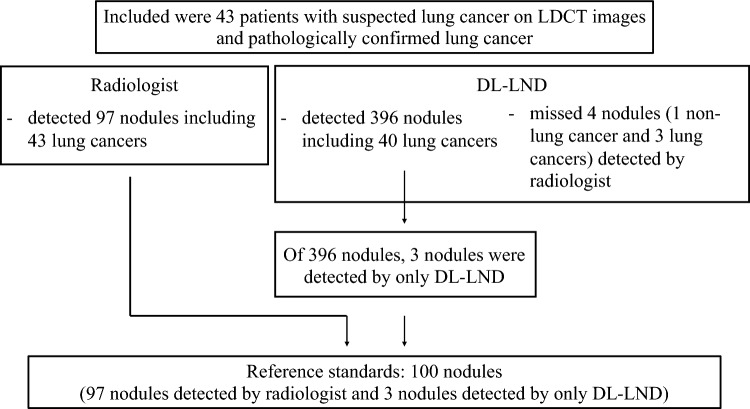
Flowchart of the reference standard nodules

**Fig. 2:**
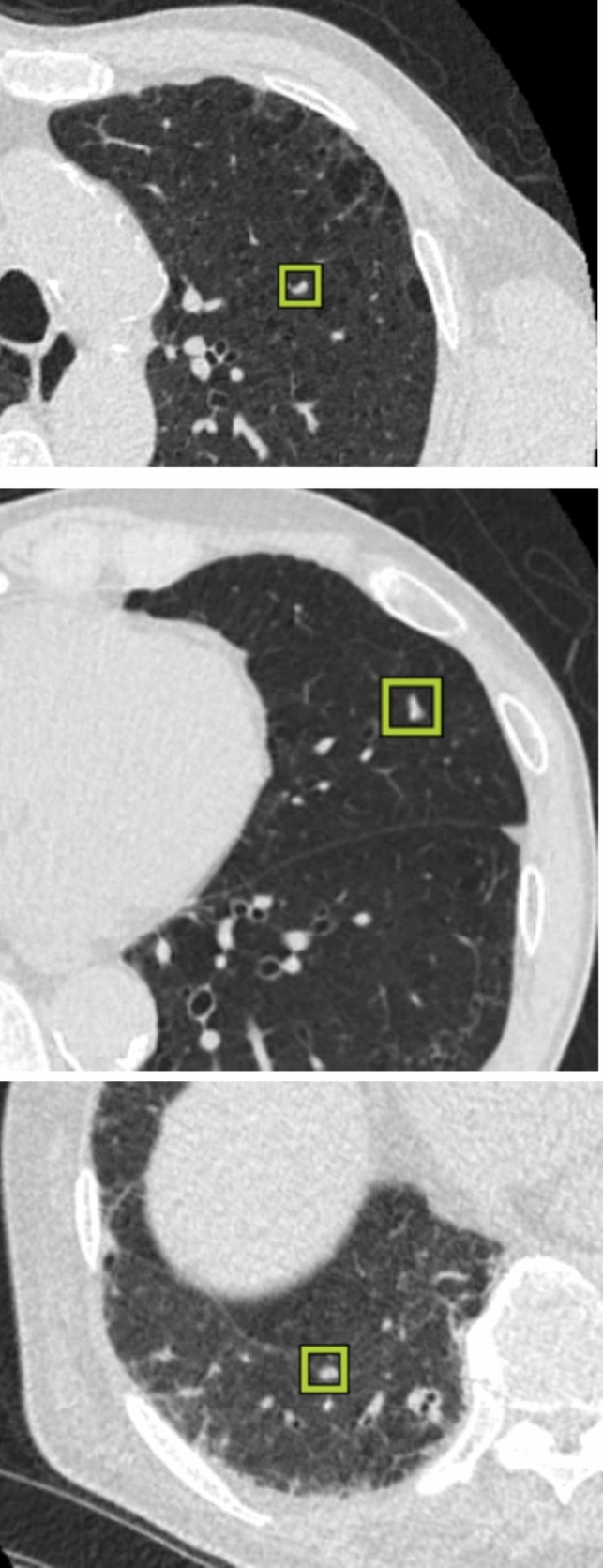
Examples of three small nodules (4 mm) detected only by DL-LND (**a**–**c**)

The radiologist detected 97 nodules (median size 8 mm, range 4–24 mm, 57 solid-, 22 GGO-, 14 part-solid GGO nodules, and 4 APC); 43 were lung cancers. He missed three small solid nodules that were detected with DL-LND. A total of 100 nodules was included in the reference standard. The flowchart for determining the reference standards for nodules is shown in Fig. 1. The three nodules missed by the radiologist are shown in Fig. 2. With respect to the location of the 100 reference nodules, 24 were in the right upper-, 17 in the right middle-, 31 in the right lower-, 17 in the left upper-, and 11 in the left lower lobe; 61 were on the lung periphery, 3 on the hilum, and 36 were in the intermediate area.

**Fig. 3:**
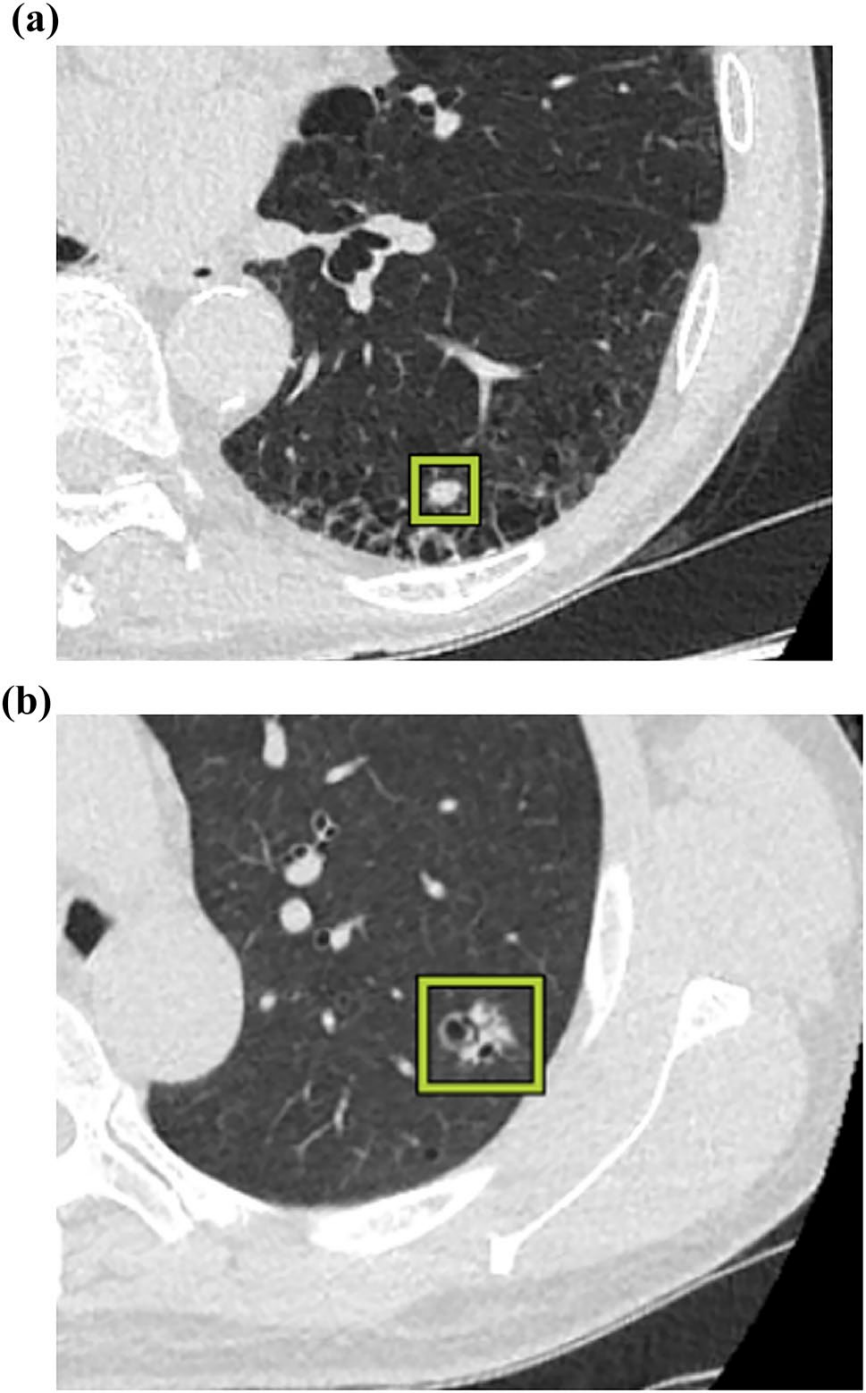
Examples of lung nodules detected by DL-LND. **a** Nodule on the interstitial pneumonia in the left lower lobe. The pathologic diagnosis was small cell carcinoma. **b** Irregular nodule with a cystic component in the left upper lobe. Surgical pathology diagnosed adenocarcinoma

**Fig. 4:**
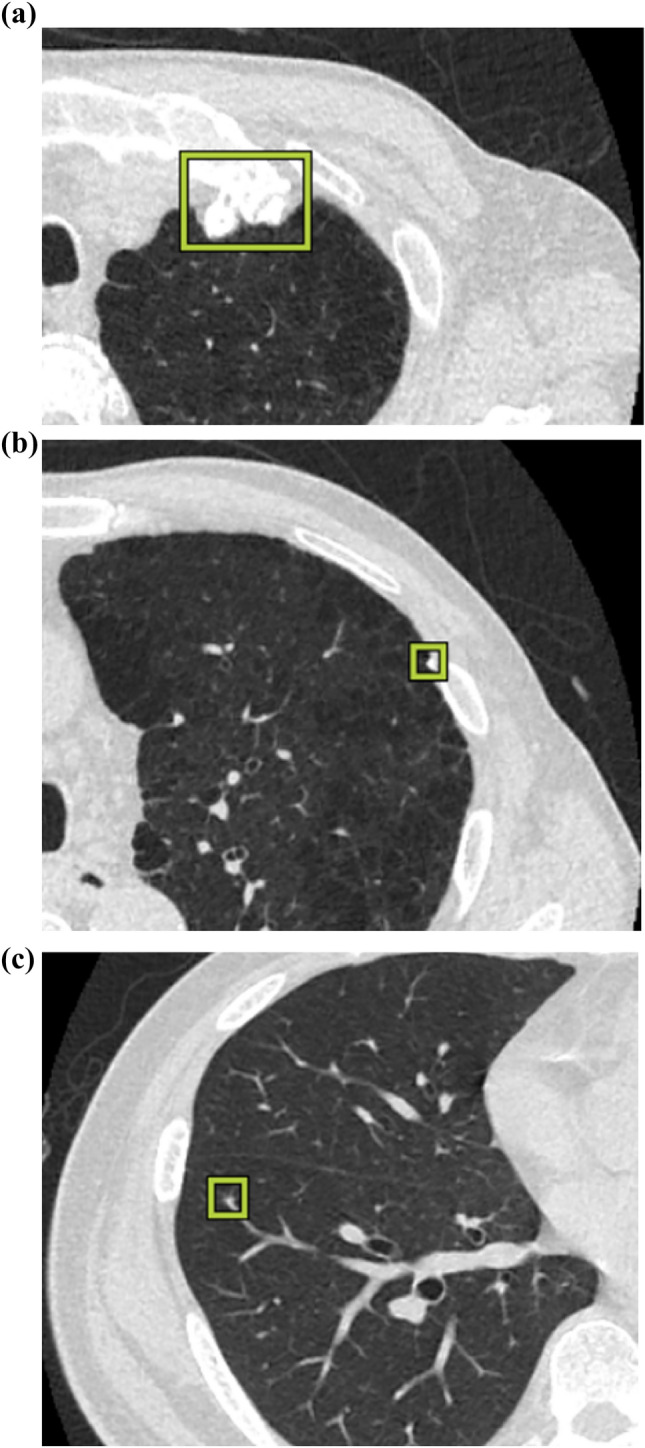
Examples of FP nodules detected by DL-LND. **a** The deformed sternoclavicular joint was mis-identified as a lung nodule. **b**, **c** Micro- and irrelevant nodules detected by DL-LND that increase the burden on radiologists

**Fig. 5:**
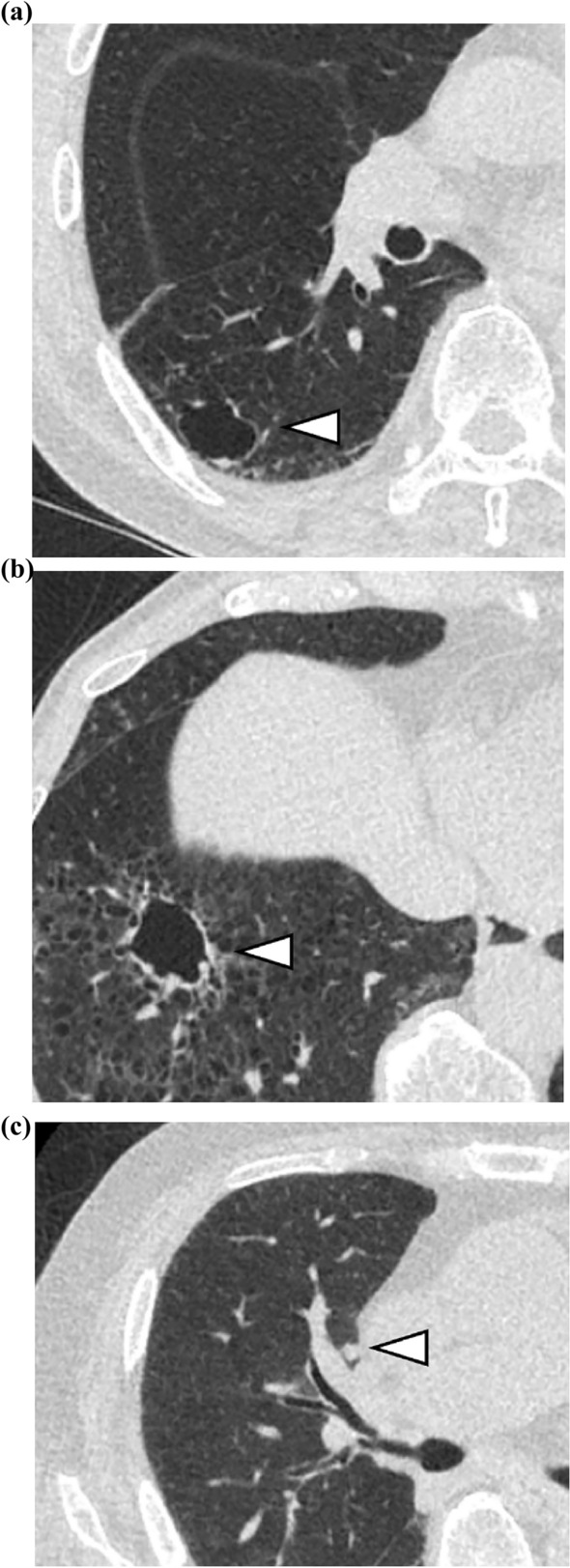
Examples of three lung cancers that were not detected by DL-LND. **a**, **b** The two missed lung cancers were atypical pulmonary cysts with thick walls and an associated nodule. They were diagnosed as squamous cell carcinoma by surgical pathology. **c** The missed lung cancer was a small nodule close to pulmonary hilum vessels. Surgical pathology identified it as a small-cell lung carcioma

DL-LND detected 396 nodules including 40 lung cancers. Its sensitivity for 100 nodules was 96.0%; the PPV was 24.2% (96/396), the mean number of FP nodules per CT scan was 7.0 (300/43). Of 300 FP nodules, 216 (72.0%) were perifissural shadows such as perifissural nodules, 79 (26.3%) were perivascular nodules such as micro-mucus plugs, and 5 (1.7%) were the sternoclavicular joint and bone spurs. Its sensitivity for lung cancer was 93.0% (40/43). DL-LND missed three lung cancers; two were APC and one was a small nodule located close to pulmonary hilum vessels. Representative cases of lung nodules detected by DL-LND and FP nodules are shown in Figs. 3 and 4. The three lung cancers missed by DL-LND are presented in Fig. 5.

## Discussion

We externally validated the performance of the commercially available DL-LND using LDCT images acquired at LCSs in Japan. Its sensitivity for lung nodules and lung cancer was 96.0% and 93.0%, respectively. Although LDCT with thin-slice images used at LCS tends to increase the image noise, thereby affecting the performance of the commercially available DL-LND, its sensitivity was high and it may prevent missing nodules. The PPV was low (24.2%) and the mean number of FP nodules per CT scan was 7.0. As the low PPV and the increased number of FP nodules require radiologists to re-read the scans, DL-LND may not reduce their reading burden.

A systematic review of AI algorithms by others who used the LIDC-IDRI database [8] revealed that their sensitivity for detecting nodules ranged from 80.0 to 96.6%. However, standard-dose CT images were used for validation and the careful external validation of commercially available DL-LND using LDCT images for LCS is needed. In the NELSON trial, Zhao YR. et al. [6] validated the performance of the commercially available Lung-CAD VB10A device (Siemens AG Healthcare). They used 400 LDCT images and found that the sensitivity was 96.7%; 1.9 nodules per scan were FP. Cui X. et al. [5] reported 90.1% sensitivity with the DL-CAD they developed and trained on the public LIDC/IDRI database; there was one FP finding on 360 LDCT images. Li L. et al. [14] evaluated the performance of the Sigma DL-CAD system (σ-Discover/lung, 12 Sigma Technologies Co. Ltd., Beijing, China); the sensitivity was 86.2% with 1.5 FP per scan on 342 LDCT images. Thus, although these AI algorithms yielded good results, their sensitivity for lung cancers, essential at LCS, remains to be validated. Besides, with the exception of Lung-CAD, not all AI algorithms are currently available in Japan.

The commercially available DL-LND validated in this study was a detection AI developed based on convolutional neural networks and trained on 1,997 chest CT images including LIDC/IDRI datasets [15]. In two public datasets its sensitivity was 99% and 96% at 5.9 and 7.3 FP nodules per scan, respectively. This DL-LND is used in more than 200 institutions in Japan although ours is the first external validation of its performance using LDCT images acquired at LCS. Our findings on the sensitivity of LDCT for lung nodule detection are consistent with those of others [5, 6, 14]. However, the mean number of FP nodules, including non-relevant small nodules per scan (7.0) was higher than that reported by others. In particular, perifissural nodules and micro-mucus plugs accounted for the majority of FP nodules. The DL-LND system we used was designed to detect solid nodules larger than 3 mm, this may have contributed to the increase in FP nodules that actually were non-relevant small nodules. The increase in FP nodules remains a considerable drawback of DL-LND since it increases the burden on radiologists.

Although DL-LND was highly sensitive for both lung nodules and pathologically confirmed lung cancer, it missed three lung cancers, 2 were APC. Approximately 0.5–9.3% of lung cancers depict a cystic component on the initial imaging scans [16] and the cancers can be missed. According to a NELSON analysis [17], 22% of missed lung cancers at the initial screening were associated with cystic spaces. Lung-RADS 2022 added new criteria for the classification and management of APC of categories 3 through 4B. Cystic features considered atypical include thick or asymmetric walls, an associated nodule, internal septations, or growth [12, 18]. Radiologists must be aware that due to insufficient training, AI algorithms may miss APC.

Overdiagnosis and overtreatment are the most significant issues in LCS [19]. They can elicit social-, psychological-, and economic problems [20]. The reference standard nodules contained 22 GGNs, 6 of which were resected, and 2 were diagnosed as adenocarcinoma in situ in our study. Although all GGNs were detected by DL-LND, their management must be considered carefully taking into account the patient’s background such as the age since they may be non-progressive or very slow-growing cancers that do not affect the prognosis.

The latest AI algorithms can not only detect lung nodules, but also analyze their characteristics and determine their benign or malignant nature. Wataya T. et al. [21] reported that DL-CAD improved the accuracy of nodular characterization and of diagnosing malignancy, especially by radiologists with less than 5 years of experience. It also increased the reproducibility of findings across radiologists. Li R. et al. [22] who reviewed deep-learning applications for lung nodule diagnosis reported that the accuracy for classifying benign and malignant lesions ranged from 75.0 to 98.2%. External verification of its performance will also be necessary in the future.

This study has some limitations. First, we only included 43 patients with pathologically confirmed lung cancer. Although we cannot deny selection bias, our findings were consistent with an earlier report that validated DL-LND using non-lung cancer cases [15]. Second, the data we validated were not large in number because the collection and analysis of a large number of cases requires an inordinate amount of time and increases the validators’ workload excessively. The construction of data sets that include LDCT images is a future challenge. Nonetheless, we think ours is the first study to externally validate the performance of the commercially available DL-LND using LDCT images at Japanese LCS. Third, our was a retrospective study performed at a single institution; it covered the period from 2015 to 2022. Due to changes in hospital regulations, the scan condition and slice thicknesses were different between 2015 (n = 11) and later (n = 32). Fourth, LDCT using thin-slice images differed from recommended DL-LND parameters. Nonetheless, DL-LND kept the high sensitivity for lung nodule detection.

In conclusion, we externally verified that the sensitivity of DL-LND for lung nodule and lung cancer detection is very high although the low PPV and the increased FP nodules remain a serious drawback.

## Abbreviations

LCS: Lung cancer screening
LDCT: Low-dose CT
NSLT: National Lung Screening
NELSON: Dutch-Belgian randomized lung cancer screening
AI: Artificial intelligence
CAD: Computer-aided detection
LIDC/IDRI: Lung Image Database Consortium and Image Database Resource Initiative
FP: False-positive
DL-LND: Deep-learning-based lung nodule detection
NCCN: National Comprehensive Cancer Network
CTDI_vol_: Computed tomography dose index
SD: Standard deviation
HU: Hounsfield units
PPV: Positive-predict value
